# Optimizing data-driven excellence: Canada’s approach to using pathogen test datasets for quality control, pipeline development and training initiatives

**DOI:** 10.1099/mgen.0.001505

**Published:** 2026-01-27

**Authors:** Kara D. Loos, Mark Horsman, Jeff Tuff, Kimia Kamelian, Darian Hole, Chanchal Yadav, Kirsten Palmier, Kristyn Burak, Molly Pratt, Connor Chato, Anna Majer, Shari Tyson, Grace E. Seo, Philip Mabon, Elsie Grudeski, Rhiannon Huzarewich, Russell Mandes, Anneliese Landgraff, Jennifer R. Tanner, Natalie Knox, Morag Graham, Gary Van Domselaar, Jessica Minion, Nathalie Bastien, Timothy Booth, Madison Chapel, Kirsten Biggar, Ana Duggan, Catherine Yoshida, Andrea Tyler

**Affiliations:** 1 National Microbiology Laboratory, Public Health Agency of Canada, 1015 Arlington Street, Winnipeg, Manitoba, R3E 3R2, Canada; 2 Roy Romanow Provincial Laboratory, 5 Research Drive, Regina, Saskatchewan, S4S 0A2, Canada; 3 Public Health Ontario Laboratory, 661 University Avenue, Toronto, ON, M5G 1M1, Canada; 4 Department of Medical Microbiology and Infectious Diseases, Max Rady College of Medicine, University of Manitoba, Winnipeg, Canada

**Keywords:** bioinformatics, genomics, quality control, SARS-CoV-2, training

## Abstract

Pathogen genomic surveillance is globally recognized as a pillar of public health. This field has expanded rapidly following the onset of the coronavirus disease 2019 (COVID-19) pandemic, and there is an urgent need to ensure the quality, comparability and reliability of the results of genomic analyses across diverse settings and analytical platforms. Currently, no methodology or framework has been universally adopted to mitigate this issue. This study aimed to provide a solution within the Canadian public health landscape by using standardized test datasets for severe acute respiratory syndrome coronavirus 2 (SARS-CoV-2) genomic analysis. In this context, a test dataset refers to a curated set of genomic sequences designed to evaluate the accuracy, consistency and performance of sequencing workflows, bioinformatics pipelines and analytical tools. These datasets serve as benchmarks, allowing laboratories to validate their methodologies and ensure comparability across different platforms. The test datasets included in this analysis were selected based on the use of well-characterized experimental protocols from the application of specimen selection criteria, through to sequence generation. Datasets generated using Illumina and Nanopore sequencing of samples from COVID-19 patients in Saskatchewan, Canada, were used and included clean controls, variable lineages and spiked-in lower-quality run data. Illumina libraries were sequenced using ARTIC network PCR amplification, while Nanopore libraries underwent similar protocols with some modifications. Public test dataset access on Zenodo further facilitates reproducibility, providing data summary outputs and pipeline environment files. A customized R script was developed to compare Illumina data, generating multiple tables and figures highlighting comparisons between analyses. The significance of this study lies in its contribution to the implementation of bioinformatic pipeline validation tools and protocols, which are essential for reliable genomic surveillance and outbreak response. By establishing a structured framework for computational validation, this study enhances the accuracy, comparability and efficiency of genomic surveillance in an evolving landscape of viral strains and testing strategies.

**Impact Statement**

Our study advances the field of pathogen genomic surveillance by introducing standardized test datasets for severe acute respiratory syndrome coronavirus 2 (SARS-CoV-2) that are well curated and freely available to the scientific community. A test dataset is a collection of known genomic sequences that laboratories can use to assess the accuracy and reliability of their analysis methods. The utility of these datasets is twofold: they provide analysts developing or updating computational pipelines with a ground truth for comparison, and they allow laboratory personnel to participate in proficiency testing to ensure they can correctly process sequencing data and handle genomic analyses. By incorporating these quality control measures into pipeline development and staff training, we have established a model that enhances the reliability and efficiency of SARS-CoV-2 surveillance. These datasets were also used in a national training workshop where participants ran their pipelines on the provided sequences and evaluated the results using the SIGNAL_output_comparison_script.Rmd. Over 90% of participants reported increased confidence in interpreting quality control metrics, highlighting the practical value of the datasets for training and capacity building. This approach strengthens quality assurance in genomic surveillance, providing a framework for maintaining best bioinformatics practices. As viral strains continue to evolve, standardized test datasets allow for adaptable and consistent testing strategies. Ultimately, our findings contribute to global efforts in improving the accuracy of SARS-CoV-2 genomic analysis and controlling the spread of the virus.

## Data Summary

The authors confirm that all supporting data, code and protocols have been provided within the article or through supplementary data files. Table S1 includes the BioSample accessions, SRA run identifiers, and associated strain IDs for all samples included in the test datasets, enabling direct traceability and facilitating reproducibility.

## Introduction

Whole-genome sequencing (WGS) has become a powerful tool in pathogen genomic surveillance, enabling rapid detection of variants, tracking of outbreaks and guiding public health interventions [1]. However, despite its widespread adoption, there remains no universally accepted framework for ensuring the comparability, reliability, and accuracy of genomic analyses across different settings and sequencing platforms. The rapid evolution of sequencing technologies, frequent software updates and differences in bioinformatics expertise across jurisdictions further complicate efforts to standardize workflows. To address this challenge, standardized test datasets have emerged as a key strategy for validating bioinformatics pipelines, training personnel and ensuring consistent results across laboratories [2].

Despite general agreement on the importance of harmonizing genomic surveillance across jurisdictions, existing public datasets used for standardization were generated as part of broader surveillance activities rather than explicitly designed as validation test datasets. As such, they often lack key features necessary for benchmarking, such as negative or mock controls, which are essential for assessing pipeline performance under realistic conditions. Additionally, many are limited to a single sequencing platform, reducing their utility for cross-platform comparisons. Variability in basecalling software, read filtering methods and primer schemes can also introduce analytical biases, complicating the interpretation and comparability of results across laboratories.

Canada provides a case study in the complexities of implementing genomic surveillance at a national scale. The Canadian healthcare system is federated, meaning that each province and territory is responsible for organizing its own patient care, including diagnostic and surveillance activities. These efforts are coordinated through Provincial Public Health Laboratories (PPHLs), which vary in their sequencing capacity, infrastructure and analytical workflows due to differences in population distribution and geography. The Canadian Public Health Laboratory Network (CPHLN), in collaboration with the National Microbiology Laboratory (NML), facilitates data sharing, provides technical expertise and develops national benchmarking standards to maintain consistency in genomic surveillance.

A key challenge in this decentralized system is ensuring that WGS and bioinformatics pipelines yield comparable and reproducible results across regions. To address this, the NML and CPHLN developed standardized test datasets for severe acute respiratory syndrome coronavirus 2 (SARS-CoV-2) genomic analysis. These datasets serve as quality control benchmarks, supporting operator training, software validation and inter-laboratory comparability. The use of test datasets is not unique to Canada—globally, similar initiatives have been undertaken, such as the US Genomics and Food Safety group’s bacterial pathogen datasets, which facilitated communication of whole genome results between jurisdictions [3]. This study builds on these efforts by establishing a framework for standardized test datasets in pathogen genomic analysis, with applications that extend beyond SARS-CoV-2 to other infectious disease surveillance programmes.

By developing structured workflows for genomic dataset validation, this study provides a model that can be adapted for bioinformatics assessment globally. As WGS becomes an integral tool for clinical and public health applications, ensuring reproducibility and standardization will be essential for outbreak response, genomic epidemiology and long-term surveillance efforts [4,5].

## Theory and implementation

### Dataset selection and upstream WGS methods

Drawing from national surveillance efforts throughout the pandemic, we curated test datasets that were designed to mimic real datasets. In keeping with the variable experimental protocols in use by different institutions, based upon operational requirements, these datasets were designed to encompass the two most widely used sequencing platforms (Illumina and ONT) and included a spectrum of possible quality variations. At the same time, consideration of data privacy was addressed by removing any identifiable metadata and simulating host contamination by artificially adding a standardized set of publicly available human sequencing reads, rather than real host data. We found that the use of these datasets was pivotal in evaluating the efficacy of our computational sequencing methodologies across varied quality scenarios and that our analysts were able to accurately interpret output results. Generating and validating these test datasets required considerable effort, reflecting a commitment to rigorous quality assurance. By doing this work centrally and making the datasets broadly accessible, we have reduced duplication of effort across jurisdictions—ultimately saving time and resources. This approach aligns with our responsibility as public health professionals to enable efficient, accurate genomic surveillance and to support the broader community in building robust analytical capacity.

#### Sample acquisition and processing

Viral RNA was extracted from nasopharyngeal swab specimens using the LuminUltra RNA Isolation Kit (cat#: RNAIK-480) in conjunction with the Kingfisher Flex Extraction system, adhering to the manufacturer’s recommendations. The detection of SARS-CoV-2 was done using a triplex multiplexed quantitative real-time PCR assay, targeting key genomic regions including the RNA-dependent RNA polymerase gene, the envelope *E* gene, alongside the human control RNase *P* gene. Methods outlined by LeBlanc *et al*. [6] were followed using specifications tailored for ABI 7500 Fast thermocyclers (Applied Biosystems). Specimens were chosen for sequencing based on a cycle threshold value less than 32, a cutoff supported by Wölfel *et al.* [7] and validated through internal experience across Canadian public health laboratories. These labs have consistently observed that sequencing success rates drop substantially above this threshold. While higher-Ct samples can be sequenced in specific contexts, such as targeted investigations, they were not included in this dataset’s general selection due to their increased likelihood of failure. Where possible, selected samples captured both dominant and minority circulating variants present during the collection timeframe [7].

#### Library preparation and sequencing

For both Illumina and Nanopore sequencing, the ARTIC network’s two-pool multiplexed PCR amplification protocol was employed, using version 1 FREED 1,200 bp primers [8,9]. Following amplification, tiled pools underwent cleanup using AMPure XP beads as per the manufacturer’s specifications. Subsequent quantification was conducted using a Quant-iT dsDNA Broad-Range Assay Kit, with input DNA quantities tailored between 100 and 500 ng for Illumina and 50 and 200 ng for Nanopore sequencing.

Illumina libraries were then prepared with Illumina Nextera DNA Flex kit and sequenced with Illumina MiSeq with Reagent kit v2 Micro (300 cycle). Sample data were then processed using the covid-19-signal-nml pipeline [10]. Nanopore libraries were prepared using Oxford Nanopore PCR tiling of SARS-CoV-2 virus with SQK-LSK109 Ligation Sequencing Kit, EXP-NBD196 Native Barcoding Expansion 96 Kit and the following modifications: (a) use of FREED primer schemes, with the volume of primer pools decreasing to 3 µl per PCR, (b) thermocycler conditions for PCR amplification set to 34 cycles as standard, (c) cDNA input was set to 200 ng per sample as input into the end-prep reaction and (d) end-prep incubation was extended to 10 min @ 20 °C and 10 min @ 65 °C. These protocol adaptations were empirically optimized and locally validated to maximize sequencing success across a wide range of sample qualities. While they diverge from earlier ARTIC v1 recommendations, such adjustments are common in public health laboratories handling clinical specimens and are not expected to introduce significant bias beyond what is typical for amplification-based approaches. Nonetheless, platform-specific limitations such as differential amplification efficiency and error profiles—particularly homopolymer-associated indels in Nanopore data—are acknowledged and accounted for in downstream analyses. Sequencing was performed on R9 FLO-MIN111 flow cells and run on the MinION Mk1C. Sequencing was terminated after 24 h, and the resulting reads were basecalled using Guppy’s v6.2.1 high-accuracy basecalling model. Pre-basecalled data was also optionally available to test out basecalling models. The resulting fastq files were then processed using the ncov2019-artic-nf connor-lab pipeline [11].

#### Illumina dataset selection

The Illumina dataset contains samples collected from March 2021 to April 2021 and September 2022 to October 2022. These samples, originating from severe acute respiratory syndrome coronavirus 2 (COVID-19) patients in Saskatchewan, Canada, were chosen to simulate varied lineage compositions across different time points. The March–April 2021 dataset reflects an early period of genomic surveillance, although limited clinical data were available due to ongoing experimentation with primer schemes across Canadian research groups. In contrast, the September–October 2022 dataset provides a supplementary set with broader lineage diversity and robust quality control metrics. To enhance the dataset’s utility, both negative and mock controls were included. The mock samples were generated by combining reads from different sources in varying proportions to emulate contamination scenarios, such as barcode bleed, cross-sample mix-ups and worst-case co-infection profiles. These proportions were intentionally selected to challenge pipeline robustness and to mimic real-world issues encountered in diagnostic and surveillance labs. Only high-quality samples with designated lineages were selected for these simulations, ensuring accurate representation of real-world sequencing challenges. This approach supports robust analyses by incorporating both temporal diversity and quality variations. Datasets are summarized in Table 1. BioSample accessions, SRA run identifiers, and associated strain IDs for all samples are available in the online Supplementary Material Table S1.

**Table 1.** Summary of Illumina and Nanopore test datasets used for SARS-CoV-2 pipeline validation and training This table outlines the key characteristics of the five standardized SARS-CoV-2 test datasets generated using Illumina or Nanopore sequencing. Datasets differ by platform, primer scheme, number of samples and controls and their intended application (e.g. proficiency testing, pipeline validation or troubleshooting). Control types include negative, mock or failed quality control (QC) samples to support a wide range of validation scenarios.

Dataset	Platform	Primer scheme	Sample	Control	Use case note
Training set A	Illumina	Freed (v1)	25	3	Early Omicron wave; baseline proficiency test set
Training set B	Illumina	Freed (v1)	25	5	Adds QC-failing negative controls to evaluate pipeline sensitivity
Training set C	Illumina	freed_V2_nml	92	5	Broader lineage diversity; for training and troubleshooting
Training set D	Illumina	freed_V2_nml	92	7	Builds on C with more mock/negative controls; suitable for tool validation
Nanopore set	Nanopore	freed_V2_nml	93	3	Used to test compatibility with long-read workflows; higher sequencing errors

#### Nanopore dataset selection

The Nanopore dataset encompassed specimens procured between September 2022 and October 2022 from COVID-19 patients in Saskatchewan, Canada. Deliberate selection yielded two distinct datasets: one characterized by exemplary controls, lineage diversity and comprehensive quality control metrics and another dataset showcasing instances of suboptimal quality control metrics (Table 1).

### Automated comparison of ncov-tools output using Illumina data

SIGNAL_output_comparison_script.Rmd, an R script developed for this study, generates a comprehensive summary report by analysing various files produced by the ncov-tools package (https://github.com/jts/ncov-tools). The script compares the performance and functionality of the new application in relation to the existing one and was specifically tailored for comparisons of Illumina data. The script begins by loading necessary packages and configurations specified in a YAML file. It then proceeds to identify and load specific files from designated folders for comparison, including ambiguous position reports, mixture reports, variant watch reports, negative control reports and quality control summary reports. Next, the script combines these data into data tables, performs necessary data manipulations and generates summary tables comparing the files’ contents between two specified folders. Additionally, the tool creates visualizations such as heatmaps and summary tables to facilitate the comparison and analysis process. Overall, the script automates the generation of a detailed summary report, aiding in the assessment and interpretation of data produced by the ncov-tools package. The script is available for access and review on our GitHub page https://github.com/phac-nml/genomic-sequencing-test-datasets. The GitHub README includes detailed instructions outlining the required directory structure, the expected input files and an example bash command for knitting the R Markdown script from the command line. Future developments aim to adapt the script for Nanopore data, thereby extending its utility across different sequencing technologies.

While ncov-tools is compatible with outputs from both ARTIC- and iVar-based workflows across Illumina and Nanopore platforms, minor differences may still arise when processing the same sample with different technologies. These discrepancies stem from differences in read length, error profiles and sequencing chemistry. For example, Nanopore data may contain homopolymer-associated indel errors not observed in Illumina data, potentially leading to variant or consensus discrepancies in low-complexity regions. Similarly, some variant calling tools interpret ambiguous base calls differently depending on platform-specific quality metrics. While this test dataset framework has not been directly benchmarked against other global platforms such as OpenEBench, it provides complementary value by emphasizing summary-level outputs like quality control (QC) metrics, watch mutations and variant calls, which are commonly interpreted by end users. We also acknowledge that containerizing the entire workflow (including ncov-tools) and embedding it in a Snakemake or Nextflow pipeline would improve usability, and this remains a priority for future development.

To support reproducibility and consistent input formatting, the SIGNAL_output_comparison_script.Rmd currently accepts only outputs from the ncov-tools pipeline, which is used by the majority of PPHLs across Canada. Users must run an upstream workflow (e.g. ARTIC or iVar) followed by ncov-tools to generate standardized outputs. The script relies on specific folder structures and filenames defined in a YAML configuration file. Limitations include potential batch effects stemming from differing lab protocols, sample selection biases (e.g. regional variant distributions) and discrepancies due to differing basecalling software versions—especially for long-read data. These factors, while generally not affecting summary-level results, may reduce reproducibility across labs. In the future, we plan to adapt the script for Nanopore data by incorporating additional filtering logic and platform-aware thresholds to address read variability and elevated error rates. We plan to expand support to non-SARS-CoV-2 pathogens by implementing schema-specific adaptations in ncov-tools, defining pathogen-specific watch mutations and developing representative test datasets. Furthermore, we intend to enhance usability and scalability by containerizing the tool and integrating it into workflow managers such as Nextflow. Collectively, these efforts will ensure broader applicability and sustained reproducibility across diverse sequencing platforms and pathogens.

### Validation strategies for SARS-CoV-2 genomic analysis

**Fig. 1.**
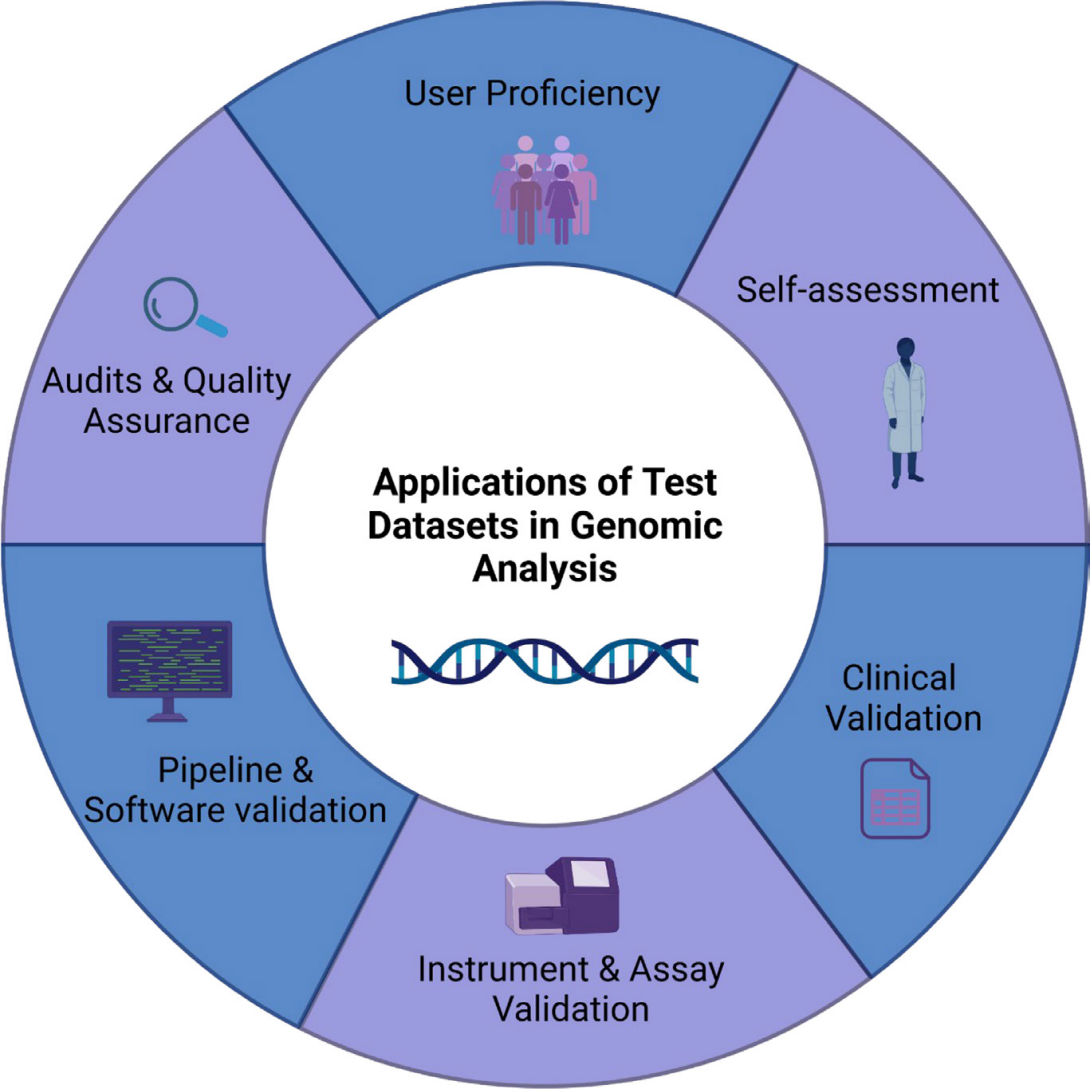
Applications of test datasets in SARS-CoV-2 genomic validation. This figure illustrates key areas where test datasets contribute to the validation of SARS-CoV-2 genomic analysis workflows. These applications include user proficiency testing to assess operator performance; self-assessment to internally verify bioinformatics and laboratory processes; clinical validation to ensure alignment with real-world conditions, using clinical samples and quality control challenges reflective of routine sequencing runs; instrument and assay validation to confirm the accuracy of sequencing platforms and diagnostic assays; pipeline and software validation to evaluate bioinformatics tools and computational workflows; and audits and quality assurance, which ensure regulatory compliance and methodological reliability. Failed datasets used in this framework were derived from samples with known quality control issues such as low depth, cross-contamination or barcode misassignment. Created using BioRender.

In the realm of SARS-CoV-2 genome analysis, ensuring the accuracy and reliability of datasets is essential. The validation process plays an important role in affirming the quality and relevance of selected datasets, particularly when using bioinformatic tools. Given the dynamic nature of the virus and its evolving lineages over time, datasets were chosen from different times throughout the pandemic to add a layer of variance to the analysis. To strengthen the validation, synthetic mock controls and biological controls have been incorporated. Synthetic controls provide standardized references for assessing tool performance, while biological controls verify the analytical process against authentic SARS-CoV-2 samples. Additionally, the inclusion of a failed dataset serves as a benchmark for identifying and addressing potential limitations, enhancing the study’s overall credibility and transparency. As illustrated in Fig. 1, test datasets are applied across multiple validation strategies, including user proficiency testing, self-assessment, clinical validation, instrument and assay validation, pipeline and software validation and audits and quality assurance. These components collectively ensure the robustness of genomic workflows by evaluating both laboratory and computational aspects of SARS-CoV-2 genome analysis. This comprehensive approach reinforces a commitment to methodological rigour, ensuring reliable genomic insights that inform public health decisions.

### Analysis interpretation guidelines

By emphasizing the stability of fundamental metrics, users can navigate potential nuances in pipeline versions and confidently draw meaningful insights from the analysis while exercising due diligence in result interpretation. Features such as depth of coverage, SNP calls and assembly completeness are critically informative to the analysis process and are sample-specific, not varying with alterations in population structure. For features which vary over the timescale of a pandemic (i.e. lineage), it is essential to exercise caution when interpreting the results derived from analysing these test datasets, as variations may arise due to changes in pipeline environment versions. While the core metrics remain robust and reliable, subtle differences in environment configurations or whether reads are dehosted could impact secondary metrics, such as lineage calls. Users are advised to prioritize the stability of key indicators rather than lineage-specific calls when assessing the findings. This awareness of potential variance underscores the importance of maintaining a standardized analytical environment and encourages a thoughtful consideration of the broader trends and patterns observed in the genomic data.

**Fig. 2.**
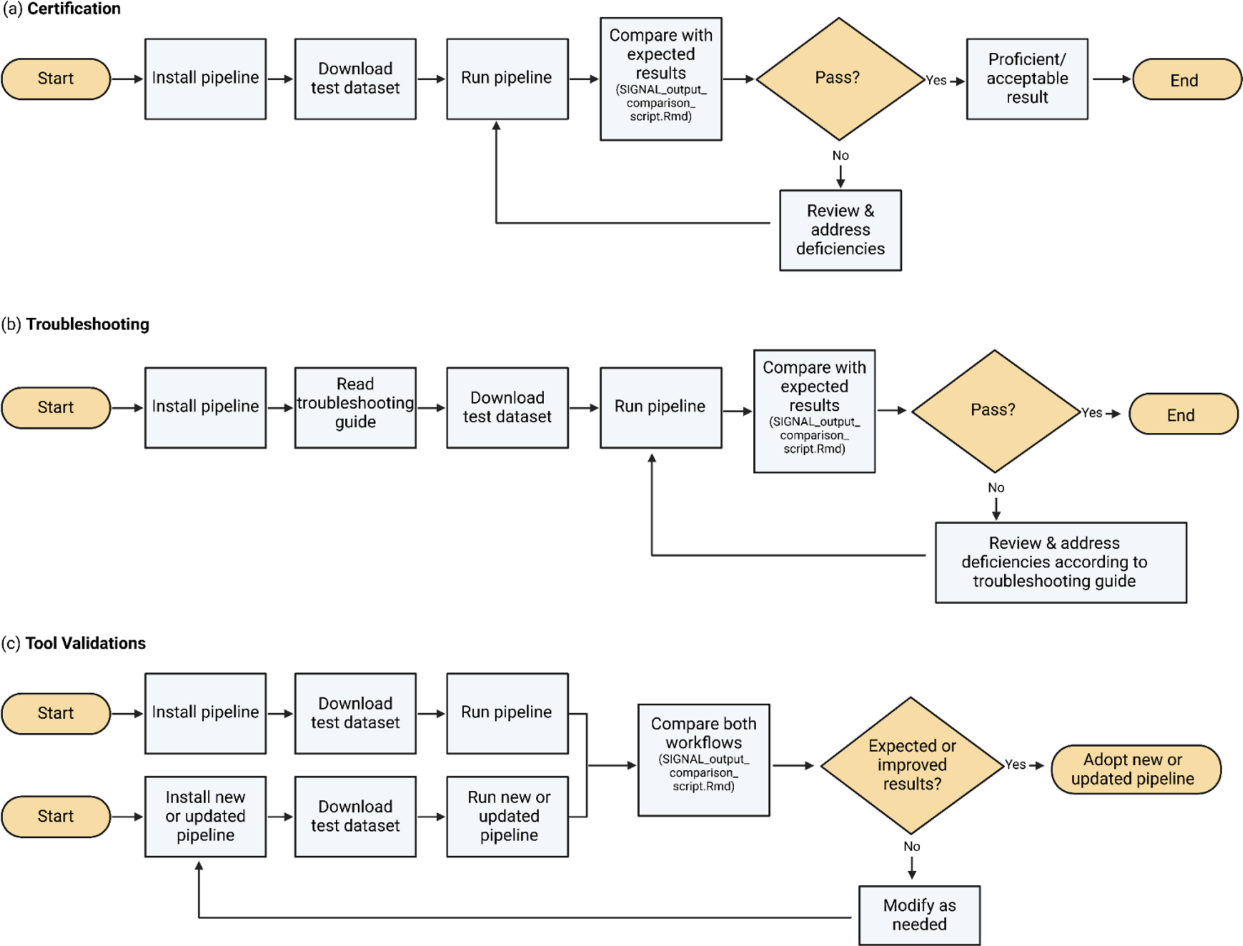
Example workflows for test dataset applications in genomic analysis. This figure outlines three structured workflows for applying test datasets in (**a**) certification, (**b**) troubleshooting and (**c**) tool validations within genomic pipelines. Created using BioRender.

**Fig. 3.**
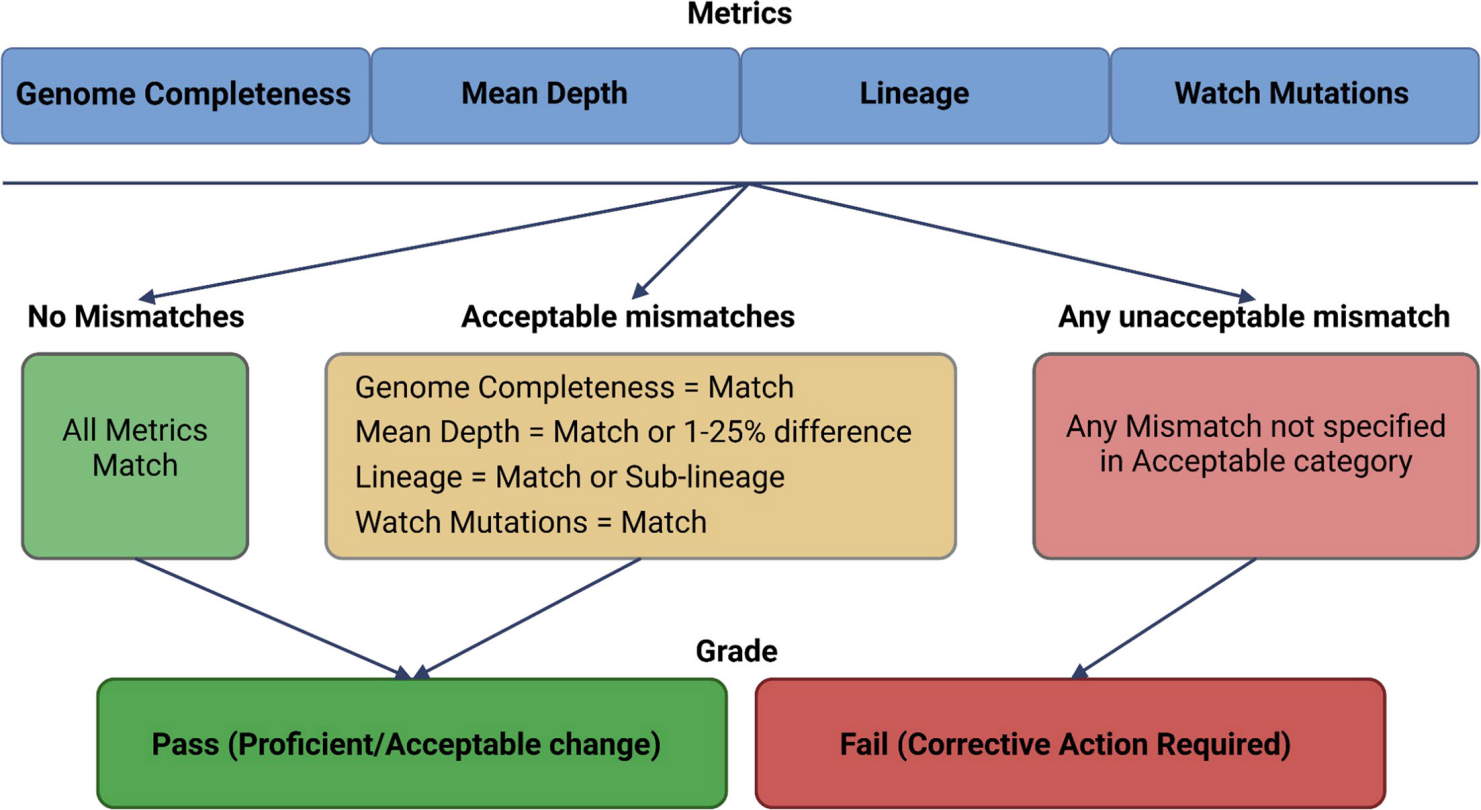
Decision framework for test dataset evaluation. The figure illustrates the decision-making process for determining the success of a test dataset application based on four key metrics: genome completeness, mean depth, lineage and watch mutations. If all metrics match expected values, the dataset is classified as proficient/acceptable (pass). If minor deviations occur within acceptable thresholds (e.g. a 1–25% difference in mean depth or a close sub-lineage classification), the dataset remains acceptable. However, the presence of any ‘Mismatch’ that does not meet acceptable criteria results in an automatic classification of corrective action required (fail). These empirically derived thresholds are intended to guide interpretation by less-experienced analysts, helping them differentiate between acceptable technical variation and true failures. For borderline results (e.g. depth just below threshold), users are encouraged to interpret conservatively and consider complementary metrics (such as completeness and watch mutation matches) before drawing conclusions. This framework ensures consistent evaluation of dataset quality while allowing for empirically derived flexibility in depth and lineage variation. Created using BioRender.

Various applications can be employed when using these test datasets, ranging from certification and training to validating updated bioinformatics tools. Fig. 2 illustrates standardized workflows for implementing these applications, ensuring a structured approach to proficiency testing, troubleshooting and validation. Users can follow these workflows to systematically assess their dataset usage and identify necessary improvements. The results obtained by adhering to these guidelines can then be evaluated using Fig. 3, which serves as a reference framework for determining whether the analysis is proficient, acceptable or requires corrective action. The 1–25% difference threshold for mean depth accommodates platform- and pipeline-specific filtering practices, such as host read removal, primer trimming and read downsampling. While not usually impacting consensus-level results, these filtering steps can cause modest depth reductions that remain within acceptable analytical limits. To support this rationale, the decision framework in Fig. 3 reflects this empirical threshold based on internal benchmarking across diverse sequencing workflows.

**Fig. 4.**
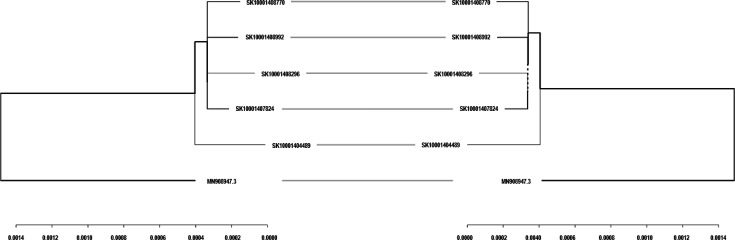
Example phylogenetic comparison generated using SIGNAL_output_comparison_script.Rmd to compare Illumina-generated SARS-CoV-2 sequence data with or without a dehosting step. The tree is midpoint-rooted for consistency and was built using consensus sequences from both preprocessing conditions. Minimal differences in tree topology and branch lengths indicate that dehosting had a negligible impact on lineage assignment. This is likely due to the high proportion of viral reads resulting from the use of PCR enrichment protocols, which selectively amplify SARS-CoV-2 RNA and reduce host-derived contamination. An entanglement score is provided to quantify tree similarity, with values ranging from 0 (identical topology) to 1 (completely mismatched).

**Fig. 5.**
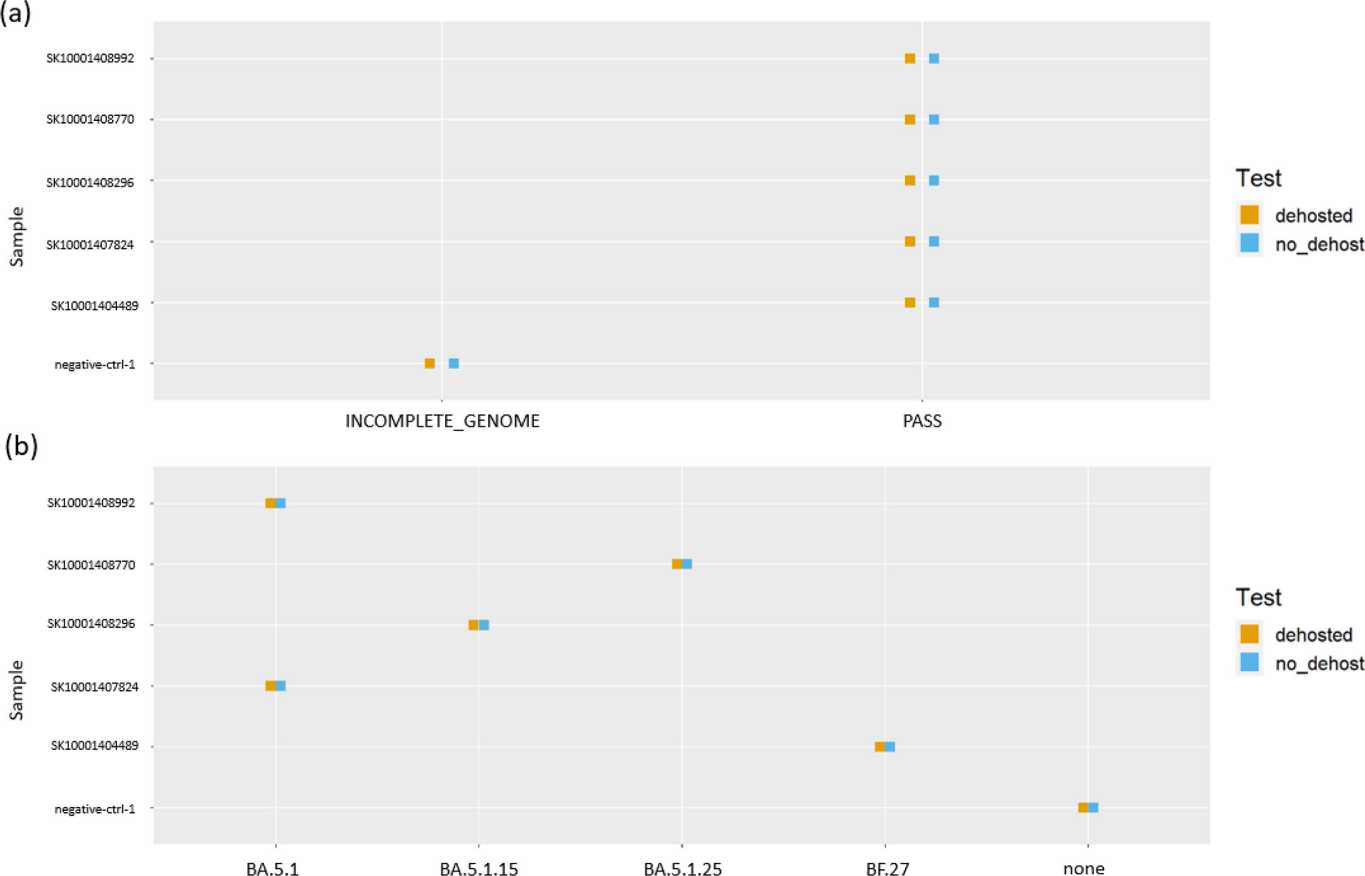
Example comparative output generated using SIGNAL_output_comparison_script.Rmd to compare Illumina-generated SARS-CoV-2 sequence data with or without a dehosting step. (**a**) Plot of QC by sample, showing consistent PASS results regardless of dehosting. (**b**) Plot of SARS-CoV-2 lineage by sample, also demonstrating consistent lineage assignment. These findings suggest that the dehosting step had minimal effect on quality control metrics and lineage determination, likely due to the high proportion of viral reads resulting from the PCR enrichment protocol used during library preparation.

As an example, SIGNAL_output_comparison_script.Rmd was used to evaluate the impact of a dehosting step on Illumina-generated SARS-CoV-2 sequence data. The script generates multiple tables and figures to visualize differences between datasets processed with and without dehosting. For this example, a selection of output figures is included in Figs4  5. Fig. 4 showcases the phylogenetic comparison output, where an entanglement score of 0 indicates a perfect match between the trees. Because dehosting involves removing host reads prior to downstream analyses, it can affect the sequences that are retained and thus alter phylogenetic tree topology—making the entanglement score a useful measure of any resulting discrepancies. Since phylogenetic trees track genome changes over time, deviations observed in this output could signal significant issues introduced by the dehosting step. Comparison plots of QC metrics and SARS-CoV-2 lineage assignments (Fig. 5a and b, respectively) show identical results between the two workflows. These plots provide valuable insights into the effects of dehosting on sequence quality, lineage classification and overall data integrity, offering a detailed assessment of the pipeline modifications under evaluation.

In addition to quantitative metrics like depth and completeness, the pipeline also evaluates mutations of interest, known as ‘watch mutations’, which are curated based on positions under surveillance by the Public Health Agency of Canada, including sites associated with variants of concern or public health relevance. While these are primarily single-nucleotide variants, the datasets may also include positions with ambiguous base calls, mixed alleles or small insertions. The ncov-tools package identifies and flags these cases in its summary reports. When such positions are encountered, ambiguous or mixed calls are either flagged or resolved using majority-based logic, depending on user-defined thresholds. The SIGNAL_output_comparison_script.Rmd incorporates these outputs into comparison tables, enabling users to assess how pipelines differ in their handling of such biologically or epidemiologically significant positions.

## Concluding remarks: advancing pathogen genomic surveillance

The comprehensive approach undertaken in this study underscores the importance of standardized test datasets and rigorous methodologies in SARS-CoV-2 genomic surveillance and outbreak response activities. By meticulously selecting datasets, implementing robust sequencing protocols and developing analytical tools, this research not only ensures the reliability and reproducibility of genomic data but also advances our understanding of virus dynamics and lineage variability. The availability of these datasets on Zenodo, accompanied by detailed data summary outputs and pipeline environment files, fosters transparency, reproducibility and collaboration within the scientific community. Moreover, the development of a SIGNAL_output_comparison_script.Rmd for comparing Illumina data and the visualization of comparison results through figures and tables further enhances the utility of these datasets for evaluating the performance of bioinformatic tools. Moving forward, continued efforts to standardize protocols, validate analytical tools and share data will be crucial for effectively combating the ongoing COVID-19 pandemic and future infectious disease outbreaks.

## Supplementary material

10.1099/mgen.0.001505 Table S1.

## Abbreviations

CPHLN: Canadian Public Health Laboratory Network
NML: National Microbiology Laboratory
PPHLs: Provincial Public Health Laboratories
QC: quality control
WGS: whole-genome sequencing
