# Supplementary material for: Optimizing data-driven excellence: Canada’s approach to using pathogen test datasets for quality control, pipeline development and training initiatives
Source: Microb Genom. 2026 Jan 27;12(1):001505. doi: 10.1099/mgen.0.001505 (PMC12847982; doi:10.1099/mgen.0.001505)
Supplement: Table S1. [file mgen-12-01505-s001.pdf]

Supplementary Table S1. Metadata table linking sequence identifiers to public database accession numbers. This table provides a mapping of sample identifiers used in the test datasets to their corresponding BioSample and SRA run accession numbers. All entries are associated with the same NCBI BioProject (PRJNA1168204). This table supports traceability and reproducibility by linking the genomic data described in the manuscript to public repositories where the raw data can be accessed.

Strain ID	BioSample Accession	SRA Run Accession
S8930	SAMN49078742	SRR33976073
S8931	SAMN49078743	SRR33976072
S8932	SAMN49078744	SRR33976047
S8933	SAMN49078745	SRR33976021
S8937	SAMN49078746	SRR33976010
S8938	SAMN49078747	SRR33975999
S8939	SAMN49078748	SRR33975988
S8940	SAMN49078749	SRR33975977
S8941	SAMN49078750	SRR33975966
S8943	SAMN49078751	SRR33976042
S8946	SAMN49078752	SRR33976071
S8947	SAMN49078753	SRR33976060
S8948	SAMN49078754	SRR33976055
S8950	SAMN49078755	SRR33976054
S8970	SAMN49078756	SRR33976053
S8971	SAMN49078757	SRR33976052
S8972	SAMN49078758	SRR33976051
S8974	SAMN49078759	SRR33976050
S8977	SAMN49078760	SRR33976049
S8978	SAMN49078761	SRR33976048
S8994	SAMN49078762	SRR33976031
S8995	SAMN49078763	SRR33976030
SK10001404489	SAMN49078764	SRR33976029
SK10001405875	SAMN49078765	SRR33976028
SK10001406173	SAMN49078766	SRR33976027
SK10001406310	SAMN49078767	SRR33976026
SK10001406311	SAMN49078768	SRR33976025
SK10001406327	SAMN49078769	SRR33976024
SK10001407423	SAMN49078770	SRR33976023
SK10001407485	SAMN49078771	SRR33976022
SK10001407595	SAMN49078772	SRR33976020
SK10001407605	SAMN49078773	SRR33976019
SK10001407677	SAMN49078774	SRR33976018
SK10001407694	SAMN49078775	SRR33976017
SK10001407749	SAMN49078776	SRR33976016

SK10001407824	SAMN49078777	SRR33976015
SK10001407832	SAMN49078778	SRR33976014
SK10001407893	SAMN49078779	SRR33976013
SK10001407912	SAMN49078780	SRR33976012
SK10001407952	SAMN49078781	SRR33976011
SK10001408002	SAMN49078782	SRR33976009
SK10001408007	SAMN49078783	SRR33976008
SK10001408049	SAMN49078784	SRR33976007
SK10001408114	SAMN49078785	SRR33976006
SK10001408116	SAMN49078786	SRR33976005
SK10001408154	SAMN49078787	SRR33976004
SK10001408285	SAMN49078788	SRR33976003
SK10001408296	SAMN49078789	SRR33976002
SK10001408316	SAMN49078790	SRR33976001
SK10001408331	SAMN49078791	SRR33976000
SK10001408357	SAMN49078792	SRR33975998
SK10001408393	SAMN49078793	SRR33975997
SK10001408430	SAMN49078794	SRR33975996
SK10001408465	SAMN49078795	SRR33975995
SK10001408482	SAMN49078796	SRR33975994
SK10001408494	SAMN49078797	SRR33975993
SK10001408498	SAMN49078798	SRR33975992
SK10001408567	SAMN49078799	SRR33975991
SK10001408580	SAMN49078800	SRR33975990
SK10001408617	SAMN49078801	SRR33975989
SK10001408651	SAMN49078802	SRR33975987
SK10001408660	SAMN49078803	SRR33975986
SK10001408662	SAMN49078804	SRR33975985
SK10001408671	SAMN49078805	SRR33975984
SK10001408673	SAMN49078806	SRR33975983
SK10001408680	SAMN49078807	SRR33975982
SK10001408696	SAMN49078808	SRR33975981
SK10001408712	SAMN49078809	SRR33975980
SK10001408731	SAMN49078810	SRR33975979
SK10001408733	SAMN49078811	SRR33975978
SK10001408739	SAMN49078812	SRR33975976
SK10001408746	SAMN49078813	SRR33975975
SK10001408766	SAMN49078814	SRR33975974
SK10001408767	SAMN49078815	SRR33975973
SK10001408770	SAMN49078816	SRR33975972
SK10001408919	SAMN49078817	SRR33975971

SK10001408958	SAMN49078818	SRR33975970
SK10001408979	SAMN49078819	SRR33975969
SK10001408981	SAMN49078820	SRR33975968
SK10001408987	SAMN49078821	SRR33975967
SK10001408992	SAMN49078822	SRR33975965
SK10001408996	SAMN49078823	SRR33975964
SK10001409004	SAMN49078824	SRR33975963
SK10001409037	SAMN49078825	SRR33975962
SK10001409041	SAMN49078826	SRR33975961
SK10001409104	SAMN49078827	SRR33975960
SK10001409120	SAMN49078828	SRR33976046
SK10001409161	SAMN49078829	SRR33976045
SK10001409163	SAMN49078830	SRR33976044
SK10001409175	SAMN49078831	SRR33976043
SK10001409200	SAMN49078832	SRR33976041
SK10001409203	SAMN49078833	SRR33976040
SK10001409220	SAMN49078834	SRR33976039
SK10001409222	SAMN49078835	SRR33976038
SK10001409299	SAMN49078836	SRR33976037
SK10001409361	SAMN49078837	SRR33976036
SK10001409397	SAMN49078838	SRR33976035
SK10001409457	SAMN49078839	SRR33976034
SK10001409465	SAMN49078840	SRR33976033
SK10001409624	SAMN49078841	SRR33976032
SK10001409698	SAMN49078842	SRR33976070
SK10001409810	SAMN49078843	SRR33976069
SK10001409859	SAMN49078844	SRR33976068
SK10001409863	SAMN49078845	SRR33976067
SK10001409890	SAMN49078846	SRR33976066
SK10001409899	SAMN49078847	SRR33976065
SK10001409907	SAMN49078848	SRR33976064
SK10001409953	SAMN49078849	SRR33976063
SK10001409979	SAMN49078850	SRR33976062
SK10001410105	SAMN49078851	SRR33976061
SK10001410106	SAMN49078852	SRR33976059
SK10001410108	SAMN49078853	SRR33976058
SK10001410169	SAMN49078854	SRR33976057
SK10001410221	SAMN49078855	SRR33976056
SK10001412070	SAMN49078050	SRR33974914
SK10001412782	SAMN49078051	SRR33974913
SK10001412806	SAMN49078052	SRR33974902

SK10001412877	SAMN49078053	SRR33974891
SK10001412914	SAMN49078054	SRR33974856
SK10001413033	SAMN49078055	SRR33974845
SK10001413138	SAMN49078056	SRR33974882
SK10001413323	SAMN49078057	SRR33974871
SK10001413355	SAMN49078058	SRR33974836
SK10001413362	SAMN49078059	SRR33974825
SK10001413391	SAMN49078060	SRR33974912
SK10001413408	SAMN49078061	SRR33974911
SK10001413553	SAMN49078062	SRR33974910
SK10001413561	SAMN49078063	SRR33974909
SK10001413681	SAMN49078064	SRR33974908
SK10001413691	SAMN49078065	SRR33974907
SK10001413766	SAMN49078066	SRR33974906
SK10001413778	SAMN49078067	SRR33974905
SK10001413783	SAMN49078068	SRR33974904
SK10001413792	SAMN49078069	SRR33974903
SK10001413802	SAMN49078070	SRR33974901
SK10001413816	SAMN49078071	SRR33974900
SK10001413836	SAMN49078072	SRR33974899
SK10001413899	SAMN49078073	SRR33974898
SK10001413917	SAMN49078074	SRR33974897
SK10001413920	SAMN49078075	SRR33974896
SK10001413947	SAMN49078076	SRR33974895
SK10001413950	SAMN49078077	SRR33974894
SK10001413965	SAMN49078078	SRR33974893
SK10001413971	SAMN49078079	SRR33974892
SK10001413977	SAMN49078080	SRR33974890
SK10001414038	SAMN49078081	SRR33974889
SK10001414126	SAMN49078082	SRR33974888
SK10001414139	SAMN49078083	SRR33974863
SK10001414142	SAMN49078084	SRR33974862
SK10001414200	SAMN49078085	SRR33974861
SK10001414201	SAMN49078086	SRR33974860
SK10001414234	SAMN49078087	SRR33974859
SK10001414235	SAMN49078088	SRR33974858
SK10001414237	SAMN49078089	SRR33974857
SK10001414248	SAMN49078090	SRR33974855
SK10001414253	SAMN49078091	SRR33974854
SK10001414282	SAMN49078092	SRR33974853
SK10001414284	SAMN49078093	SRR33974852

SK10001414296	SAMN49078094	SRR33974851
SK10001414303	SAMN49078095	SRR33974850
SK10001414545	SAMN49078096	SRR33974849
SK10001414586	SAMN49078097	SRR33974848
SK10001414606	SAMN49078098	SRR33974847
SK10001414648	SAMN49078099	SRR33974846
SK10001414652	SAMN49078100	SRR33974844
SK10001414747	SAMN49078101	SRR33974843
SK10001414753	SAMN49078102	SRR33974842
SK10001414770	SAMN49078103	SRR33974841
SK10001414792	SAMN49078104	SRR33974840
SK10001414798	SAMN49078105	SRR33974887
SK10001414800	SAMN49078106	SRR33974886
SK10001414812	SAMN49078107	SRR33974885
SK10001414815	SAMN49078108	SRR33974884
SK10001414879	SAMN49078109	SRR33974883
SK10001414899	SAMN49078110	SRR33974881
SK10001414926	SAMN49078111	SRR33974880
SK10001414998	SAMN49078112	SRR33974879
SK10001415004	SAMN49078113	SRR33974878
SK10001415062	SAMN49078114	SRR33974877
SK10001415099	SAMN49078115	SRR33974876
SK10001420888	SAMN49078116	SRR33974875
SK10001421128	SAMN49078117	SRR33974874
SK10001421134	SAMN49078118	SRR33974873
SK10001421240	SAMN49078119	SRR33974872
SK10001421246	SAMN49078120	SRR33974870
SK10001421253	SAMN49078121	SRR33974869
SK10001421254	SAMN49078122	SRR33974868
SK10001421256	SAMN49078123	SRR33974867
SK10001421342	SAMN49078124	SRR33974866
SK10001421343	SAMN49078125	SRR33974865
SK10001421357	SAMN49078126	SRR33974864
SK10001421370	SAMN49078127	SRR33974839
SK10001421371	SAMN49078128	SRR33974838
SK10001421438	SAMN49078129	SRR33974837
SK10001421440	SAMN49078130	SRR33974835
SK10001421458	SAMN49078131	SRR33974834
SK10001421460	SAMN49078132	SRR33974833
SK10001421472	SAMN49078133	SRR33974832
SK10001421474	SAMN49078134	SRR33974831

SK10001421476	SAMN49078135	SRR33974830
SK10001421482	SAMN49078136	SRR33974829
SK10001421483	SAMN49078137	SRR33974828
SK10001421486	SAMN49078138	SRR33974827
SK10001421569	SAMN49078139	SRR33974826
SK10001421591	SAMN49078140	SRR33974824
SK10001423134	SAMN49078141	SRR33974823
SK10001423135	SAMN49078142	SRR33974822
